# Influence of Australasian Triage Scale on time to CT for major trauma patients: a Western Australian level 1 trauma centre experience

**DOI:** 10.1007/s00068-025-02989-2

**Published:** 2025-10-28

**Authors:** Kyle Raubenheimer, Jeni Thomas, Gabriel Plitzko, Joseph Trimboli, Pradeep Sanjamala, Dieter G. Weber

**Affiliations:** 1 Royal Perth Hospital, Perth, Australia; 2 University Medical Center Hamburg-Eppendorf, Hamburg, Germany; 3 University of Western Australia, Perth, Western Australia; 4 Trauma Services, Department of General Surgery, Royal Perth Hospital, 197 Wellington St, Perth, WA 6000 Australia

**Keywords:** Major trauma, Triage, Time to CT, Australasian Triage Scale

## Abstract

**Purpose:**

This study investigated whether higher triage urgency according to the Australasian Triage Scale (ATS) is associated with reduced time to computed tomography (CT) in major trauma patients.

**Methods:**

A retrospective analysis was conducted on 621 patients with major trauma (ISS > 12) admitted to the state adult major trauma centre in Western Australia between September 2021 and August 2022. Demographic, injury, and clinical data were extracted from the state trauma registry. Time to CT was analysed using multivariate regression to assess the influence of ATS category and other clinical variables.

**Results:**

Median age was 50 years (range 14–103), and 75% were male. Nearly half of patients were triaged as ATS category 1. Higher urgency ATS category was independently associated with shorter time to CT: compared with ATS category 1, time to CT was delayed by 39 min in ATS 2 and 91 min in ATS 3 patients (*p* < 0.001). Male sex was also associated with faster time to CT (− 27 min, *p* < 0.001). Longer time to CT correlated with increased time in the emergency department but was not associated with in-hospital mortality.

**Conclusion:**

Higher ATS urgency predicts faster access to CT in major trauma. Female patients experience longer delays, which may warrant targeted interventions. Time to CT was not associated with mortality in this cohort.

## Introduction

Computed tomography (CT) is a critical imaging modality in modern trauma care. Expedited CT in major trauma is associated with reductions in time to diagnosis, time in the emergency department (ED), and laparotomies, as well as improvements in other metrics associated with good clinical care [1–4]. ED length of stay has been shown to be an independent predictor of mortality for trauma patients [5].

In Australia, patients presenting to the ED are triaged according to the Australasian Triage Scale (ATS) [6]. The ATS is a 5-level clinical tool predicated on a brief history and clinical assessment typically performed by trained triage nurses used to establish maximum waiting time for patients (Table 1) [6]. The ATS is applied prospectively with the aim to facilitate timely emergency care [6]. Since major trauma has been shown to impact staff workload within the ED, appropriate triaging facilitates effective management of major trauma patients, and contributes to the overall quality of care through the appropriate allocation of resources [7].

**Table 1:** The Australasian triage scale (ATS) [6]

ATS Category	Level of Urgency	Time to Treatment Goal	Clinical Examples
1 - Immediate	Life-threatening	Immediate (0 min)	Cardiac arrest, severe trauma, unconscious patient, massive bleeding
2 - Emergency	Imminent risk of deterioration	Within 10 min	Severe respiratory distress, major burns, suspected stroke, acute chest pain (possible MI)
3 - Urgent	Potentially serious	Within 30 min	Moderate asthma, significant head injury, moderate bleeding, severe abdominal pain
4 - Semi-Urgent	Less serious, but requires attention	Within 60 min	Mild asthma, minor fractures, stable but painful injuries
5 - Non-Urgent	Least serious, can wait	Within 120 min	Mild cold/flu symptoms, chronic issues without acute distress, minor wounds

The Royal Australasian College of Surgeons trauma verification criteria recommends data collection on time to CT as a quality indicator for Australasian trauma systems [8, 9]. International guidelines recommend the time to CT from arrival to ED to be less than 1 h for severely injured patients [10, 11], while others have suggested more aggressive time protocols [12–14]. Previous studies in Australia have demonstrated a time to CT of between 43 min and 76 min [15, 16]. However, the potential influence of the triage category may impact the urgency in which patients in the ED receive their CT scans.

While it is intuitive that patients with higher triage priority receive expedited CT imaging, quantifying this relationship is crucial for evaluating the efficiency of trauma care delivery. Delays in CT imaging can result in prolonged diagnostic uncertainty, delayed interventions, and worse clinical outcomes in critically ill trauma patients [4, 17]. Additionally, examining the time to CT across different ATS categories can provide insights into whether triage-based prioritization aligns with actual patient needs and clinical outcomes, or whether systemic inefficiencies exist that disproportionately affect specific patient subgroups [18]. For instance, prior studies have demonstrated that workflow inefficiencies, resource limitations, and interdepartmental communication barriers can contribute to unexpected delays in imaging, even for high-acuity patients [19].

The primary aim of this study was to quantify the time to CT for major trauma patients at the state adult major trauma centre in WA over a 1-year period and to investigate the effect of ATS triage category on time to CT. Understanding this relationship is critical, as adherence to time-to-CT benchmarks has been linked to improved trauma outcomes, including reduced morbidity and mortality [20, 21]. Furthermore, this study seeks to determine the effect of time to CT on time in ED, the relationship between time to CT and mortality, and to determine other variables associated with time to CT in Western Australia.

## Methods

### Study population

All patients with major trauma, defined as Injury Severity Score (ISS) > 12, who were admitted to Royal Perth Hospital and recorded in the Western Australian State Trauma Registry between 01 September 2021 and 31 August 2022 were analysed. Inclusion in the registry requires definitive treatment within 7 days of trauma, hospital admission longer than 24 h, or trauma-related death. Interhospital transfers were included in the study, provided that CT scans were performed at Royal Perth Hospital.

### Setting

Royal Perth Hospital is the designated adult major trauma centre for the State of Western Australia and accepts all major trauma patients within the State, over the age of 14 years. All adult major traumas within the State of Western Australia are channelled through the regional trauma network to Royal Perth Hospital for definitive care. Royal Perth Hospital employs a ‘trauma call’ system, where prehospital criteria such as mechanism of injury, physiology, and physician discretion determine ATS category designation. Computed tomography (CT) imaging is available at all times, with an in-house radiologist present 24 h a day, 7 days a week. Requesting of CT scans is performed either in person or by telephone consultation with the on-duty radiologist.

For the purpose of this study, ‘metropolitan area’ refers to the Perth Metropolitan Region as defined by the Western Australian Metropolitan Region Scheme (MRS), which covers approximately 6,400 km² and extends around 90 km north to south and 40 km inland from the coast.

### Data collection

Data for this study were obtained from the Western Australian State Trauma Registry. Variables extracted included time to CT, ATS category, time of patient triage, time first seen by a member of the medical team, and time of first recorded CT image. In addition, patient demographics (age and sex), mechanism of injury, injury severity score (ISS), and hospital admission details (including time in ED, ED disposition destination, hospital length of stay [LOS], and final discharge destination), as well as in-hospital mortality, were collected from the registry.

Time to CT was defined as time from emergency department triage to time of first images recorded on the hospital imaging system (IMPAX, Agfa-Gevaert Group, Mortsel, Belgium). For time of first image recorded, time of first scouting image is recorded as an automated process. Where a first scouting image is not loaded onto the hospital imaging system, the manually recorded time on the first image on the hospital system was recorded.

### Study approval

Approval from the hospital Clinical Safety and Quality Unit was obtained prior to commencement of this study (GEKO Approval #49617). Study was conducted according to the STROBE guidelines [22].

### Statistical analysis

Initial exploratory data analysis was performed and outliers were removed from the dataset. A threshold of 8 standard deviations was used to remove outliers (11 outliers were removed from the 632 original study patients).

QQ-plots and Kolmogorov-Smirnov test was utilized to assess the normal distribution of the data. Normally distributed data were compared using a two-tailed unpaired t-test, whereas non-normally distributed and ordinal-scaled data were compared using the Mann-Whitney U test. Differences between nominal data were analyzed with the χ² (chi-squared) test. If the event count was below five, Fisher’s exact test was applied.

For time to CT and mortality, univariate regression was used to identify significant variables to include in separate multivariate regression models. Hospital length of stay, ED disposition, and outcome were included in exploratory analyses but were not primary predictors of time to CT, as they occur post-imaging. Statistical significance was set at *p* < 0.05. Statistical analyses and data visualisations were completed using R Statistical Package (R Foundation for Statistical Computing, Vienna, Austria) [23].

## Results

### Demographics

Between 01 September 2021 and 31 August 2022, 621 major trauma patients underwent CT imaging. The median age was 50 years (range 14–103), and most patients were male (*n* = 464, 75%). The most common mechanisms of injury were falls (*n* = 188, 30%), motor vehicle crashes (*n* = 144, 23%), motorbike crashes (*n* = 96, 15%), being struck by objects (*n* = 45, 7%), and pedal cyclist incidents (*n* = 34, 5%). A total of 457 patients (74%) sustained their injuries in the metropolitan area. Nearly half of the cohort were triaged as ATS category 1 (*n* = 302, 49%). Between ATS categories, there were statistical differences in biological sex, rurality, time to CT, time in ED, ED disposition, and final discharge destination. Patients with higher urgency ATS category had a greater proportion from metropolitan areas, were more often admitted to the ICU, and were less frequently discharged directly home. Overall, the most common discharge destination was home (*n* = 361, 58%), followed by rehabilitation facilities (*n* = 100, 16%). The median Injury Severity Score (ISS) was 19 and the median hospital length of stay (LOS) was 9 days (Table 2).

**Table 2:** Descriptive statistics of the study population

Variable	Category/Measurement	Total	ATS 1	ATS 2	ATS 3	ATS 4/5	*p* value^†^
		*n* = 621	*n* = 302	*n* = 210	*n* = 104	*n* = 5	
Sex	Male	464 (75%)	226 (75%)	165 (79%)	70 (67%)	3 (60%)	< 0.001***
	Female	157 (25%)	76 (25%)	45 (21%)	34 (33%)	2 (40%)	
Age	Median, years (IQR)	50 (32–67)	46 (29–61)	51 (35–67)	63 (44–77)	73 (71–86)	0.064
	Range	14–103	14–95	15–99	15–103	71–87	
Rurality	Metropolitan	457 (74%)	209 (69%)	155 (74%)	88 (85%)	5 (100%)	< 0.001***
	Rural/Remote	164 (26%)	93 (31%)	55 (26%)	16 (15%)	0 (0%)	
Clinical Variables	GCS, median score (IQR)	15 (14–15)	15 (13–15)	15 (14–15)	15 (15–15)	15 (15–15)	0.42
	HR, median BePM (IQR)	85 (73–100)	89 (74–105)	84 (74–98)	77 (66–92)	72 (64–76)	0.817
	SBP, median mmHg (IQR)	130 (117–148)	123 (106–140)	136 (125–151)	138 (124–153)	144 (109–147)	0.667
ISS	Median, score (IQR)	19 (16–26)	24 (17–30)	17 (14–24)	17 (14–21)	16 (14–17)	0.998
	Range	13–75	13–75	13–75	13–43	14–22	
Time to CT	Median, minutes (IQR)	108 (75–174)	83 (64–116)	146 (90–188)	218 (142–281)	237 (148–361)	0.002**
	Range	3–555	3–518	31–528	9–555	118–368	
Time in ED	Median, minutes (IQR)	282 (192–386)	209 (158–306)	312 (228–398)	394 (308–504)	505 (437–568)	0.03*
	Range	105–1578	21–1053	91–1098	140–1260	333–651	
ED Disposition	Operating Theatres	46 (7%)	34 (11%)	11 (5%)	1 (< 1%)	0	< 0.001***
	ICU	103 (17%)	90 (30%)	13 (6%)	0	0	
	SMTU High Acuity	111 (18%)	67 (22%)	33 (16%)	10 (10%)	1 (20%)	
	SMTU Low Acuity	260 (42%)	98 (32%)	107 (51%)	54 (52%)	1 (20%)	
	Other Wards	101 (16%)	13 (5%)	46 (22%)	39 (38%)	3 (60%)	
Discharge Destination	Home	361 (58%)	151 (50%)	136 (65%)	71 (68%)	3 (60%)	< 0.001***
	Rehabilitation Facility	100 (16%)	77 (25%)	21 (10%)	2 (2%)	0	
	Hospital Transfer	60 (10%)	21 (7%)	24 (11%)	15 (14%)	0	
	Death	48 (8%)	27 (9%)	13 (6%)	8 (8%)	0	
	Discharge at own risk	23 (4%)	14 (5%)	8 (4%)	1 (1%)	0	
	Residential Care Facility	11 (2%)	2 (< 1%)	1 (< 1%)	6 (6%)	2 (40%)	
	Other	18 (3%)	10 (3%)	7 (4%)	1 (1%)	0	
LOS	Median, days (IQR)	9 (4–18)	12 (6–25)	7 (3–14)	4 (3–9)	7 (5–9)	0.995
	Range	0–145	0–145	0–130	1–43	1–15	

^**†**^ Statistical analysis only conducted for ATS categories 1–3 due to low numbers in ATS categories 4–5

*ATS* Australasian Triage Scale, *IQR* Interquartile Range, *GCS* Glasgow Coma Scale, *HR* Heart Rate, *BeBPM* Beats Per Minute, *SBP* Systolic Blood Pressure, *DBP* Diastolic Blood Pressure, *RR* Respiratory Rate, *BrBPM* Breaths Per Minute, *ISS * Injury Severity Score, *ED* Emergency Department, *LOS* Length of Stay

## Influence of triage category on time to CT

**Fig. 1:**
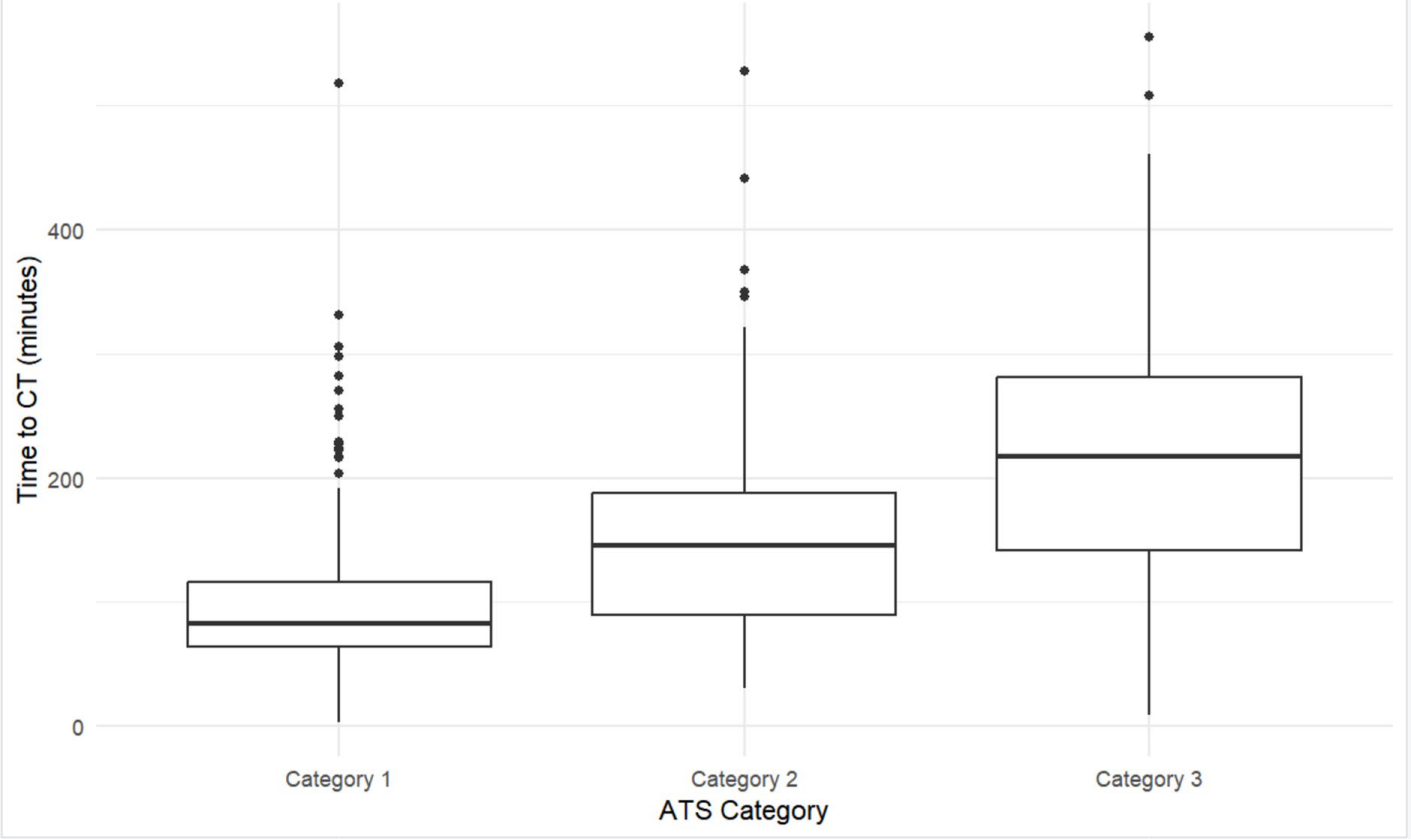
Boxplots of Time to CT by Australasian Triage Category

Comparisons of the demographics between different triage categories are described in Table 2. Higher urgency ATS category was associated with a faster time to CT (Fig. 1). Using ATS category 1 as a reference point, patients who were triaged as ATS category 2 received a CT scan 39 min later, whilst patients triaged as ATS category 3 received CT scans 91 min later. Due to low numbers, patients in ATS category 4 and 5 were not included in the analysis. Notably, inclusion of outliers did not change statistical significance of main analysis.

### Time to CT and time in ED

**Fig. 2:**
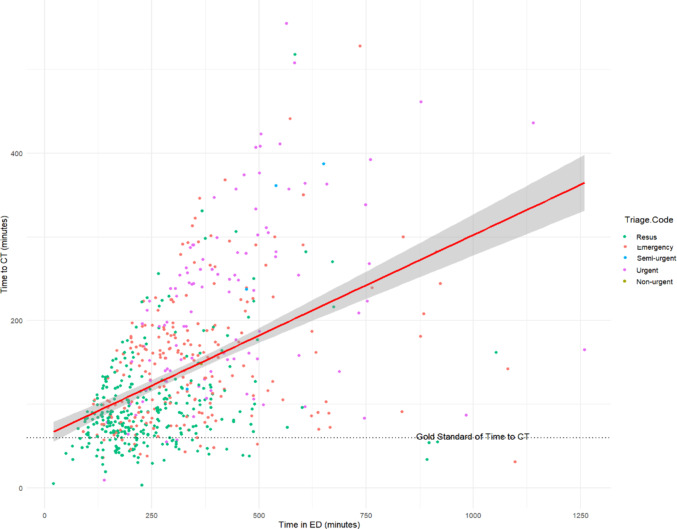
Scatterplot of Time to CT vs. Time in ED by Australasian Triage Category Score

Increases in time to CT were associated with increased time in ED (Fig. 2). In both the univariate and multivariate modelling, time to CT was correlated with time in ED (Tables 3 and 4). Multivariate regression analysis determined that for every 1-minute increase in time in ED, there is an increased time to CT of 9 s (Table 4). The intercept for the multivariate model was 51 min for time to CT.

**Table 3:** Univariate analysis of variables associated with time to CT

Variable	β_1_	Adjusted *R*^2^ Value	*p* value
ISS	−1.981	0.046	< 0.001***
Age	0.635	0.021	< 0.001***
Gender (ref: Female)		0.019	< 0.001***
Male	−28.672		< 0.001***
TBI Category (ref: Mild)		0.044	< 0.001***
Moderate	−25.966		0.073
Severe	−70.075		< 0.001***
Arrival Temperature	−0.133	−0.002	0.801
Arrival HR	−0.328	0.004	0.058
Arrival SBP	0.513	0.023	< 0.001***
Arrival DBP	0.306	0.001	0.174
Arrival RR	−1.169	0.003	0.116
Rurality (ref: Country)		0.002	0.12
Metropolitan	12.424		0.12
ATS Category (ref: Category 1)		0.277	< 0.001***
Category 2	55.079		< 0.001***
Category 3	126.917		< 0.001***
Mechanism	125.429	0.015	0.073
Time in ED	0.235	0.221	< 0.001***
ED Disposition (ref: SMTU Low Acuity)		0.093	< 0.001***
ICU	−67.001		< 0.001***
Operating Theatres	−52.983		< 0.001***
SMTU High Acuity	−36.574		< 0.001***
Other Wards	5.808		0.556
Length of Stay	−0.779	0.02	< 0.001***
Outcome (ref: Death)		0.06	< 0.001***
Home	34.893		0.008**
Hospital Transfer	51.092		0.002**
Non-Hospital Transfer	−14.232		0.681
Own Risk	8.625		0.691
Rehabilitation Facility	−10.215		0.497
Residential Care Facility	68.403		0.027*
Other	−1.486		0.962

*ISS * Injury Severity Score, *TBI* Traumatic Brain Injury, *HR* Heart Rate, *SBP* Systolic Blood Pressure, *DBP* Diastolic Blood Pressure, *RR* Respiratory Rate, *ATS* Australasian Triage Scale, *ED* Emergency Department, *SMTU* State Major Trauma Unit, *ICU* Intensive Care Unit

**Table 4:** Multivariate regression model of variables associated with time to CT

Variable	Estimate	Standard Error	*p* value
ISS	−0.109	−0.281	0.778
Age	0.100	0.575	0.565
Gender (ref: Female)			
Male	−26.998	−3.885	< 0.001***
TBI Category (ref: Mild)			
Severe	−16.799	−1.114	0.266
Arrival SBP	0.188	1.529	0.127
ATS Category (ref: Category 1)			
Category 2	39.080	5.325	< 0.001***
Category 3	91.238	9.281	< 0.001***
Time in ED	0.155	8.312	< 0.001***
ED Disposition (ref: SMTU Low Acuity)			
ICU	−4.972	−0.383	0.702
Operating Theatres	−8.670	−0.692	0.489
SMTU High Acuity	−11.971	−1.403	0.161
Length of Stay	0.242	0.987	0.324
Outcome (ref: Death)			
Home	9.94	0.671	0.502
Hospital Transfer	11.081	0.66	0.51
Residential Care Facility	0.991	0.035	0.972

*ISS * Injury Severity Score, *TBI* Traumatic Brain Injury, *SBP* Systolic Blood Pressure, *ATS* Australasian Triage Scale, *ED* Emergency Department, *SMTU* State Major Trauma Unit

### Time to CT and mortality

Multivariate analysis did not show any significant difference in time to CT on mortality (*p* = 0.410). When time to CT was included in a multivariate model, factors associated with mortality included ISS (*p* < 0.001), age (*p* < 0.001), moderate or severe TBI (*p* < 0.001), systolic blood pressure (*p* = 0.014), diastolic blood pressure (*p* = 0.025), ATS category 3 (*p* = 0.014), length of stay (*p* = 0.014), and ED disposition to the ICU (*p* = 0.022).

### Variables associated with time to CT

Univariate modelling determined that ISS, age, sex, severe TBI, arrival SBP, ATS category, time in ED, length of stay, ED disposition, and outcome were statistically significant variables associated with time to CT (Table 3). Statistically significant variables of the univariate analysis were used for the multivariate model. Statistically significant variables associated with reductions in time to CT included male gender (*p* < 0.001), moderate TBI (*p* = 0.032), and ED disposition to other wards (*p* = 0.003). Variables that reached statistical significance and were associated with increased time to CT included ATS categories 2 and 3 (*p* < 0.001) and time in ED (*p* < 0.001) (Table 4).

## Discussion

This series demonstrates a clear association between a higher urgency ATS triage category with a reduction in the time to CT. To our knowledge, this is the first time the relationship between time to CT and triage category has been defined. Other notable observations include a description of the demographics of WA major trauma patients receiving CT scans, lack of evidence on an effect of time to CT and mortality, and the variables associated with time to CT at our centre.

The result of reduced time in ED associated with reductions in time to CT is in keeping with the current literature. Time to CT has been shown to reduce time in ED within New Zealand, and elsewhere [24, 25]. Since CT scanning provides the basis for many diagnoses, expedited CT scanning leads to faster time to diagnosis and thus expedited disposition from the ED [17]. Whilst studies have investigated the time to the CT itself, fewer studies have commented on the time to CT report – a crucial step that many non-radiological clinicians rely on prior to deciding on an appropriate disposition for non-critical injuries. In-house emergency radiologists have been shown to reduce report turnaround significantly [25]. Future studies may consider incorporating the temporal variable of report turnaround time to gain a better understanding of factors related to time to CT, time in ED and clinical outcomes.

The present study did not find a statistically significant effect of time to CT on mortality for major trauma patients. This observation is in keeping with the Australian literature and the only multicentre, randomised controlled trial for time to CT [15, 16]. However, recent studies have postulated that faster time to CT may be associated with reductions in mortality, with times less than 30 min shown to have reduced mortality [13, 17, 26]. Furthermore, certain injuries are more likely to benefit from an expedited time to CT than others [26], suggesting patient selection and optimisation of in-hospital trauma systems may be important. Time to CT of less than 26 min was associated with lower mortality in blunt traumatic injury, whilst reduced time to angioembolization (which typically proceeds following a positive CT at our centre), has been shown to reduce mortality in pelvic trauma patients [26, 27]. In contrast, despite time to craniectomy having been shown to improve mortality in a military cohort, time to CT head does not seem to influence mortality in TBI [22, 24, 28, 29]. The absence of an observed effect of time to CT on mortality in this study is likely explained by sample size, cohort heterogeneity, and the overall low mortality rate in the WA trauma system. Moreover, our analysis included all CT scans across major trauma presentations, whereas certain subgroups (e.g. blunt aortic injury or pelvic trauma) may derive greater benefit from expedited imaging [26]. Organisational and infrastructural factors of trauma centres may also influence time to CT [29]. Future studies should stratify patients by specific injury patterns to better determine which cohorts benefit most from reduced imaging delays.

Several variables were associated with time to CT when in the multivariate regression modelling. Male gender, moderate TBI and disposition from the ED to inpatient wards other than the State Major Trauma Unit ward resulted in reduced time to CT. The negative association with admission to the State Major Trauma Unit ward is likely a marker of sicker patients, with a higher multi-system involvement of their traumatic injuries. Conversely, longer time in the ED and lower urgency ATS category were associated with longer times to CT. The biological sex discrepancy may be partially explained by delays in obtaining pregnancy test results prior to CT scans for potentially pregnant patients. However, this alone may not fully account for the discrepancy, as major trauma activations typically prioritize urgent imaging when clinically indicated. Other factors, such as differences in injury patterns, triage decisions, or implicit biases in clinical workflows, may warrant further investigation. Moderate, but not severe TBI, was associated with decreased time to CT as compared to mild TBIs. This may be the result of the interventions required for severe TBI, particularly as a GCS less than 8 typically warrants intubation. Time to sedation for patients following RSI has been shown to be between 12 and 55 min, which may account for the lack of statistical significance in the multivariate regression model for severe TBIs [30]. The decision for expeditious imaging in unwell patients should be balanced against the time requirement for interventions. In patients with a high degree of suspicion of severe TBI, expeditious imaging may facilitate timely transfer to the operating theatre for urgent cranial decompression. If the intervention is not lifesaving, it may be prudent to obtain imaging to facilitate faster diagnosis and subsequent interventions that may improve outcomes. ED disposition to other wards may indicate the opposite, as less complex traumatic injuries may not require as comprehensive scanning strategies, resulting in faster CT scans and subsequent dispositions to specialty wards.

We acknowledge several limitations in this study. This retrospective case-series was performed at a single centre. Whilst data for the trauma registry is collected prospectively, the research question is retrospective and prone to bias associated biases and methodological limitations. As data was only collected from a single centre, external validity of the study to other centres with other environments, hospital policies and procedures. We acknowledge that certain variables have low sample sizes, potentially resulting in exaggerated statistical significance without concurrent clinical significance. Finally, we note that trauma team activations (TTAs), time-to-provider, and ATS category are not independent variables but overlapping components of the same urgency framework. At Royal Perth Hospital, a two-tiered TTA system operates in parallel with ATS assignment, with higher urgency ATS categories frequently triggering a Tier 1 activation. Similarly, the time from arrival to first medical assessment is directly determined by ATS thresholds. For this reason, ATS was selected as the primary measure in our analysis, as it is a standardised, externally validated triage system applied universally across emergency presentations, whereas TTA criteria and time-to-provider are centre-specific and inherently coupled to ATS urgency. While this overlap limits the ability to isolate the independent contribution of TTA or time-to-provider, our approach ensures comparability and generalisability of findings to other trauma systems that also apply ATS or similar validated triage scales.

## Conclusion

Higher urgency ATS triage category is associated with shorter time to CT for major trauma patients. Shorter time to CT was also associated with reduced time spent in the emergency department. Female patients experienced longer delays to CT compared with male patients.

## Data Availability

The data underlying this article are not publicly accessible due to their sensitive character. They are, however, available from the corresponding author on reasonable request. The data are kept in a controlled-access data storage at Royal Perth Hospital.
